# Polycomb group protein Suz12 is regulated by a novel miRNA-like small RNA

**DOI:** 10.1038/s41598-018-19989-5

**Published:** 2018-01-29

**Authors:** Patrice Penfornis, Joseph D. Fernandes, Radhika R. Pochampally

**Affiliations:** 1 Cancer Institute, University of Mississippi Medical Center, Jackson, MS 39216 USA; 2 Department of Biochemistry, University of Mississippi Medical Center, Jackson, MS 39216 USA

## Abstract

Human mesenchymal stem/stromal cells (hMSCs) provide support for cancer progression, partly through their secretome that includes extracellular vesicles (EVs). Based on deep-sequencing of small RNA from EVs of MSCs, we now report the characterization of novel small RNA, named n-miR-G665, which exhibits typical properties of miRNAs. n-miR-G665 sequence is conserved and expressed in most cell types. Knockdown studies using anti-agomirs and shRNA studies demonstrated that n-miR-G665 plays an important role in cell proliferation. Functional assays to reveal the targets of n-miR-G665 showed that polycomb protein Suz12 is regulated by n-miR-G665, which in turn regulates the expression of n-miR-G665 through feedback loop mechanism. These data shed light on a previously unknown novel feedback regulatory mechanism for controlling Suz12 expression regulated by previously not described miRNA, which may highlight a new therapeutic approach to control the polycomb repressor complex 2 activity in cancers.

## Introduction

Deep sequencing of human transcriptome offers an unbiased approach that has been used over the past few years not only to identify differences in expression levels at RNA but also allowed discoveries of novel types and small regulatory RNAs (less than 200 nucleotides) including and not limited to microRNA (miRNA), piwi-interacting RNA (piRNA), small interfering RNA (siRNA), small nucleolar RNA (snoRNAs), tRNA-derived small RNA (tRF), small rDNA derived RNA (srRNA) and Y-RNAs^1^. All the small non-coding RNAs vary in their function, structure and mechanisms^2^. Furthermore, the technique is used to identify novel small RNA candidates that are lowly abundant or poorly conserved but still relevant in the biological or physiological context^3,4^.

Human mesenchymal stem/stromal cells (hMSCs) are plastic adherent cells derived from bone marrow, referred commonly as marrow stromal cells and later classified as multipotent mesenchymal stromal cells^5^. Various studies have shown that hMSCs act as stromal cells for solid tumors where they localize, integrate into the tumor associated stroma^6,7^. Once integrated, apart from providing stromal support, hMSCs promote tumor growth and angiogenesis through juxtacrine, paracrine and endocrine mechanisms^8,9^. However the underlying mechanism by which hMSCs support tumor growth remains largely unexplored.

Extracellular vesicles (EVs) are the secreted small membrane vesicles that form intracellular multi-vesicular compartments and are released upon fusion of these compartments with the plasma membrane. EVs include exosomes (40–100 nm diameter), shedding microvesicles (50–1,000 nm), and apoptotic bodies (50–5000 nm). EVs exosomes represent a specific subtype of secreted vesicles that has been described to transport proteins, lipids, mRNAs, small RNAs, and small molecule metabolites^10,11^. Further, the transfer of functional small RNAs have been demonstrated between neighbor cells^12,13^. As such, these EVs are increasingly considered paracrine effectors with a broad spectrum effects on targeted cells^14,15^.

Polycomb group proteins are known to regulate the chromatin structure^16^. Polycomb repressive complex 2 (PRC2) catalyzes the methylation of histone H3 at lysine 27 conferring its role as an epigenetic regulator^17^. PRC2 complex is composed of four core subunits: EZH2 (Enhancer of zeste homolog 2), EED (embryonic ectoderm development protein), SUZ12 (Suppressor of zeste 12 protein homolog) and RBBP7/4 (retinoblastoma-binding protein 7/4). The impact of PRC2 complex in the regulation of gene-expression has been demonstrated during early embryo development and it is conserved in eukaryotes, from plants to flies to humans^16,18^.

In this study, based on the deep sequencing and bioinformatics analysis, we found a novel candidate miRNA/short/small RNA and tested to demonstrate that the novel small RNA is a miRNA (n-miR-G665). The novel miRNA-like was validated in secondary structure, quantitative and qualitative expression, target gene analysis, and biological functions. We provide functional evidence that n-miR-G655 targeted PRC2 recruitment via binding to SUZ12 3′UTR mRNA sequence. Broadly, our work identifies a novel short RNA that regulates cell proliferation and may form an auto-regulatory loop.

## Results

### Prediction and characterization of novel miRNA-like RNA identified in the deep sequencing data

**Figure 1:**
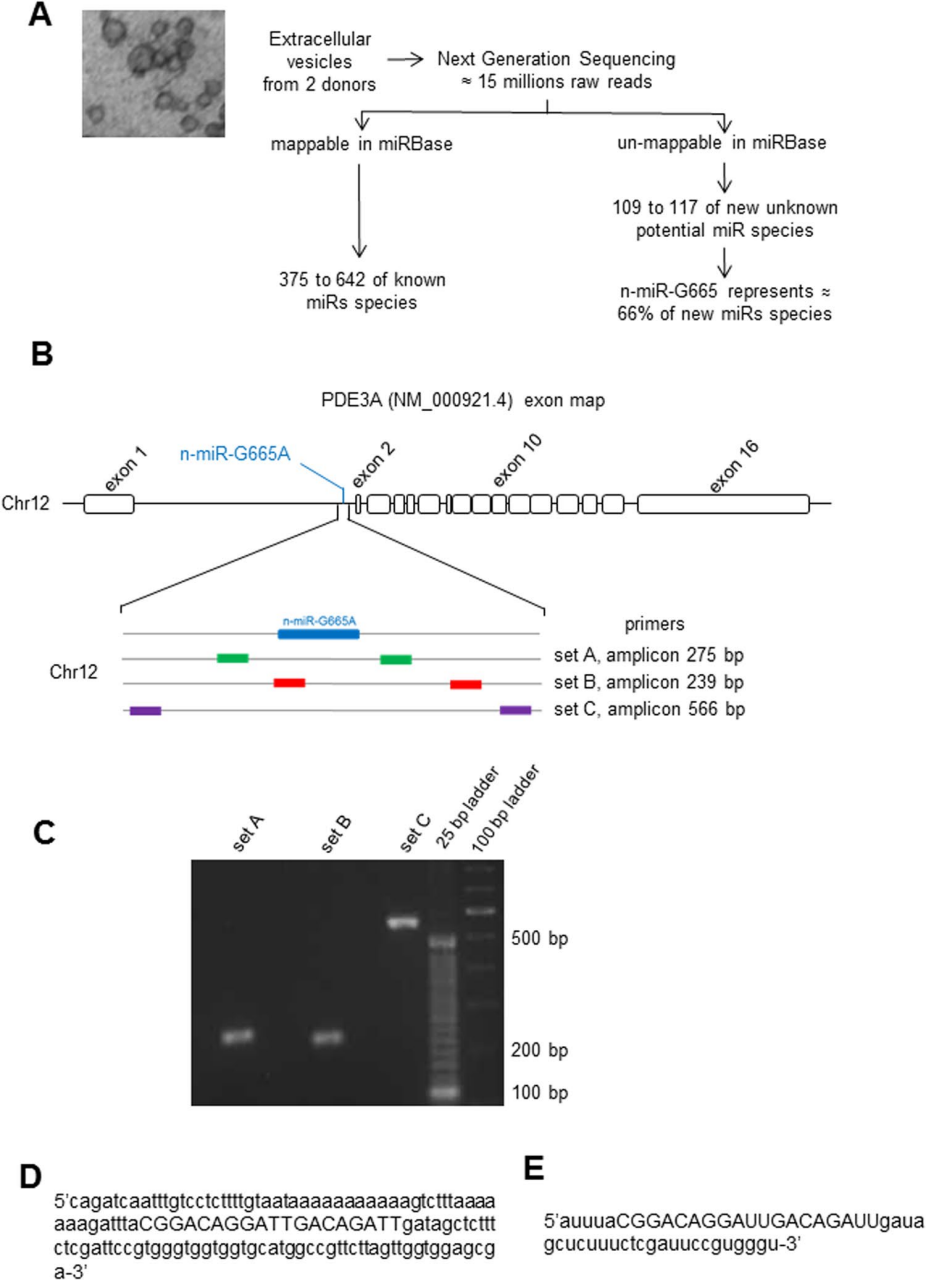
NGS detection and location of n-miR-G665: (**A)** Flow chart of new miRNA species detection strategy by next generation sequencing (NGS). (**B)** Representation of intron-exon of PDE3A gene and location of n-miR-G665 sequence on intron 1. Schematic of primers sets used to covers n-miR-G665 genomic location and amplicons size expected (**C)** Verification of predicted amplicons band size on agarose gels. (**D)** Sequence of the genomic location of n-miR-G665 (in capital letters) and (**E)** RNA sequence of n-miR-G665 (in capital letters).

We have analyzed RNA-Seq data published previously by our laboratory^19^ to search for novel sequences with predicted miRNAs sequence in EVs secreted by hMSCs. From over 15 million raw reads, we have identified 109–117 new or yet unreported short RNA species. Of those, one potential miRNA constituted 66% of new miRNA-like RNA species on total copy number reported (Fig. 1A and Supplemental Figure S1). The following was the sequence prediction of pre-miRNA-like gene: 5′cagatcaatttgtcctcttttgtaataaaaaaaaaaagtctttaaaaaaagatttaCGGACAGGATTGACAGATTGATAGCtctttctcgattccgtgggtggtggtgcatggccgttcttagttggtggagcga-3′ (potential mature miRNA-like gene sequence is capitalized) (Fig. 1D). A BLAST analysis (http://www.ncbi.nlm.nih.gov/BLAST/) of candidate miRNA-like sequence has located it on human chromosome 12, strand+, from 20551369 to 20551503 (UCSC: GRCh38 Genome Reference Consortium Human Reference 38, ID: GCA_000001405.15) (Fig. 1B). It is in the first intronic region of PDE3A gene between exon 1 and exon 2. Position and amplicons sizes have been confirmed using quantitative PCR assays with a set of primers that covers regions containing the novel miRNA-like gene sequence (Fig. 1B,C).

**Figure 2:**
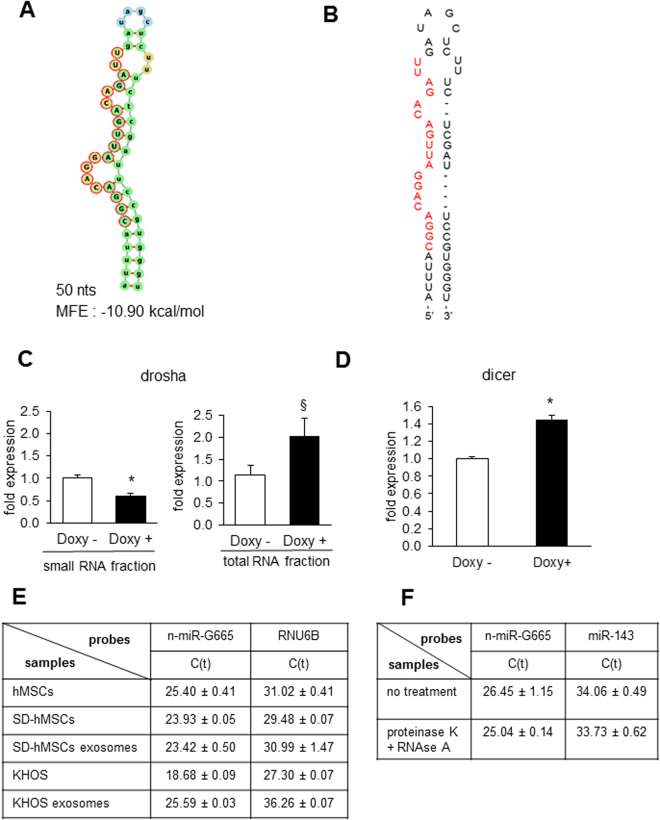
Potential secondary structure of n-miR-G665, processing and expression in cells and extracellular vesicles: (**A**) Minimum free energy (MFE) and potential secondary structure of pre-n-miR-G665 sequence obtained with RNAFold (green: stems, yellow: interior loops and blue: hairpin loop, NGS detected sequence circled in red). (**B**) Schematic representation of pre-n-miR-G665 based on the optimal thermodynamic ensemble prediction (NGS detected sequence in red). (**C**) Depletion of n-miR-G665 in small RNA fraction in Drosha −/− cells and enrichment in total RNA fraction, ^§^*P* < 0.05, *P < 0.01. (**D**) n-miR-G665 is dependent of Dicer (total RNA fraction, *P < 0.01) (**E**) Expression of n-miR-G665 in normal MSCs, serum-deprived MSCs and from exosomes purified from SD-MSCs, compare to KHOS cancer cells and exosomes purified from KHOS. (**F**) n-miR-G665 in SD-MSCs exosomes after proteinase K and RNAse treatments, miR143 is used as a EVs containing miRNA control.

miRNAs originate from longer, non-protein coding RNAs and they have an ability to form hairpins with miRNA sequence in one of its arms^20^. Based on a 50 nucleotides sequence of n-miRNA (Fig. 1E), we used the RNAFold web server (http://rna.tbi.univie.ac.at/cgi-bin/RNAWebSuite/RNAfold.cgi) to obtain a prediction of a potential secondary structure with a minimum free energy of −10.90 kcal/mol (Fig. 2A,B). Secondary structure and relative high MFE obtained led us to conclude that this new small RNA has miRNA-like features and we named it n-miR-G665.

### n-miR-G665 is Drosha and Dicer dependent

One important characteristic of miRNA processing is Drosha and Dicer dependent maturation of pre-miRNA. Next we tested the expression levels of n-miR-G655 following Drosha and Dicer knockdown. We used doxycycline regulated model in hMSCs that were described previously^21^. Cells were treated with doxycycline, then short and total RNA fraction were extracted using differential precipitation as described in Methods section. Quantitative PCR assays demonstrated that the expression of n-miR-G665 is reduced in the small RNA fraction and enriched in the total RNA fraction (Fig. 2C), this suggest that n-miR-G665 required Drosha for its maturation/processing. Using an *in vitro* model of Dicer knockout cells, we observed that n-miR-G665 expression in total RNA fraction is increased when Dicer was knocked out (Fig. 2D), corroborating the Drosha knockdown studies finding and confirming that n-miR-G665 is processed through canonical miRNA maturation pathway, further supporting that the small RNA is a miRNA. Since, we observed n-miR-G665 expression in EVs, produced by serum deprived hMSCs, we tested EV enrichment in EVs as opposed to parental cells using q-PCR studies (Fig. 2E). This EVs enrichment seems to be distinct for hMSCs cells since it is not observed in a cancer cell line model (KHOS) (Fig. 2E). To confirm that n-miR-G665 were contained and protected into extracellular vesicles, we treated EVs successively with proteinase K and RNase A prior to RNA isolation and observed no significant changes in its expression suggesting that n-miR-G665 was not free floating in suspension as protein–RNA complexes but effectively associated to extracellular vesicles (Fig. 2F).

### Phylogenetic conservation of n-miR-G665 expression

**Figure 3:**
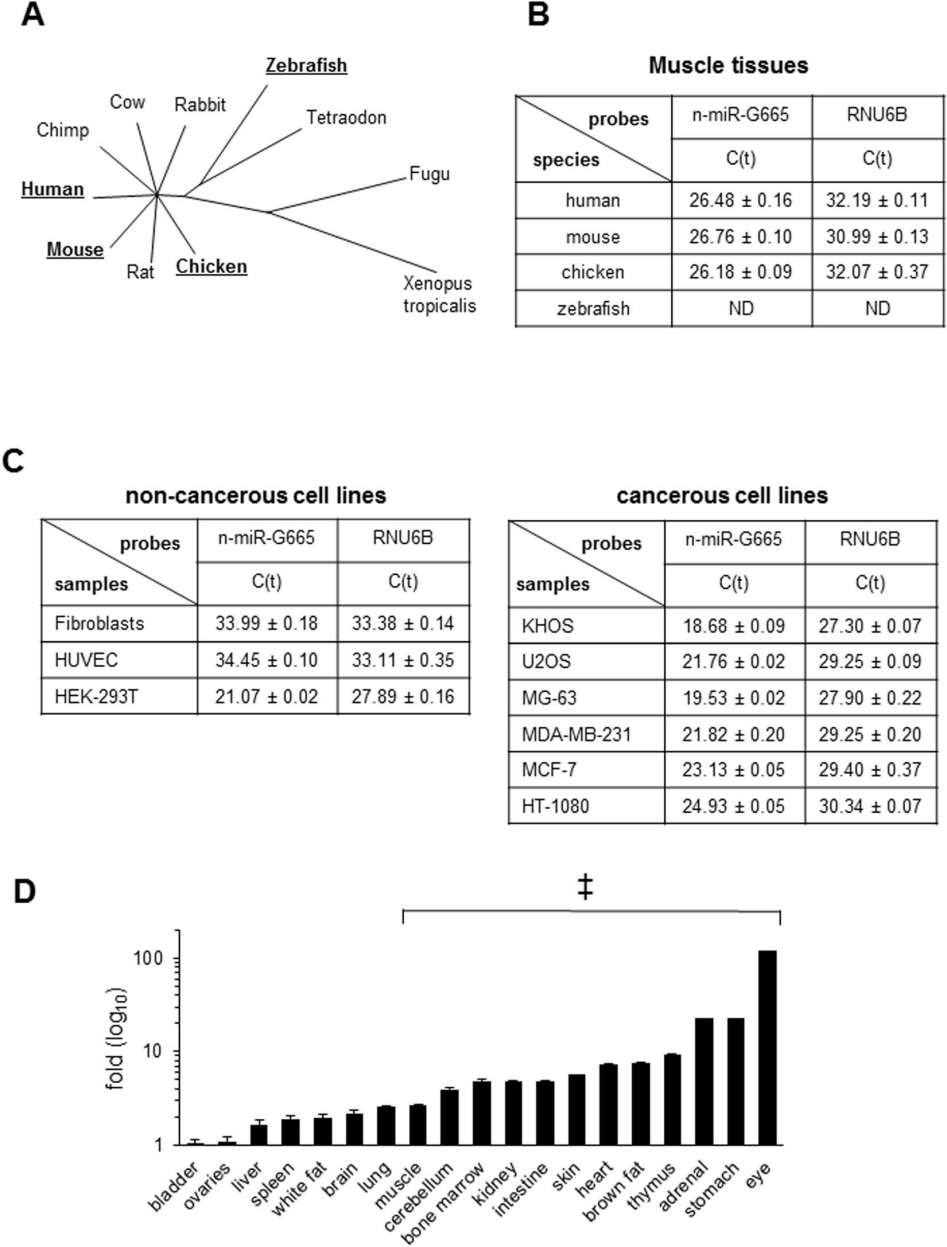
n-miR-G665 differential expression and tissue distribution. (**A**) Radial phylogenic tree representation based on sequence alignment of n-miR-G665 in UCSC Genome Browser on Human May 2004 (NCBI35/hg17) Assembly. (**B**) Expression of n-miR-G665 in muscular tissue of different vertebrate species, ND: not detected. (**C**) Expression of n-miR-G665 in various normal and cancerous cell lines. (**D**) Relative expression in normal *nu/nu* female mouse tissues, ^‡^above 2-fold increase.

Next logical step was to identify the expression of this miRNA both vertically and horizontally in evolutionary pathway. Based on sequence alignment in gene ontology studies, the radial phylogenic tree of n-miR-G665 demonstrates 100% conservation in mammals (Fig. 3A and Supplemental Figure S2). The phylogenetic tree associations were further confirmed using q-PCR assays using muscle tissue of representative members of the species (Fig. 3B). Further, we tested the expression in various mouse tissues and while all tissues expressed n-miRNA G665, certain tissues such as bladder, ovaries or liver contained significantly less amount of n-miR-G665 compared to thymus, stomach or eye tissues (Fig. 3D). Next, we tested the expression and distribution of n-miR-G665 in *in vitro* cell line models using both cancerous and non-cancerous models (Fig. 3C) demonstrated a variable expression as well.

### n-miR-G665 knockdown impairs cell proliferation and embryo development

**Figure 4:**
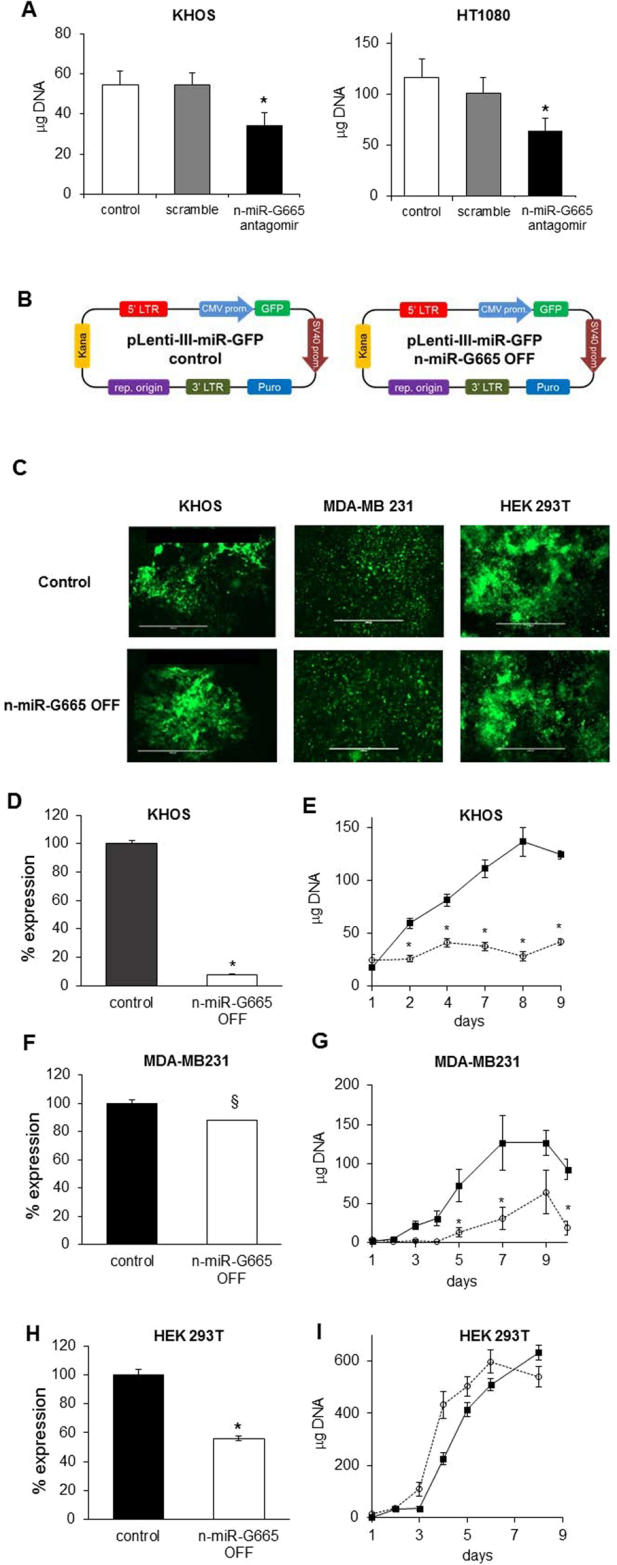
n-miR-G665 knock-out expression impairs cell proliferation *in vitro*. (**A**) Effect of transient downregulation of n-miR-G665 on cell growth, *P < 0.01. (**B**) Lentiviral transfection models used and (**C**) Representative pictures of GFP-expressing transfected cell lines obtained. (**D**,**F** and **H**) Relative n-miR-G665 expression in control and n-miR-G665 OFF cell lines (^§^*P* < 0.05, *P < 0.01), and (**E**,**G** and **I**) growth curves of control (plain square) and n-miR-G665 OFF (open circles) respective cell lines, *P < 0.01.

Next, we investigated the functional role of n-miR-G665 using different cancer cell lines. We observed that the transient transfection of an inhibitory sequence of n-miR-G665 (anti-agomiR) in mesenchymal tumors models (KHOS and HT1080) impeded their proliferation rate/growth (Fig. 4A). We generated stable knock out of n-miR-G665 of mesenchymal (KHOS) and epithelial tumor cell line models (MDA-MB-231 and HEK-293T) using a lentiviral based shRNA (Fig. 4B,C). Interestingly, a 90% decrease of n-miR-G665 expression impaired the proliferation of KHOS cell line dramatically (Fig. 4D,E). The proliferation of MDA-MB-231 cell line was moderately reduced, which can be explained by less than 10% reduction of n-miR-G665 expression (Fig. 4F,G). The dramatic effect on proliferation was not as pronounced in HEK-293T cell line despite a 45% decrease of n-miR-G665 expression (Fig. 4H,I).

**Figure 5:**
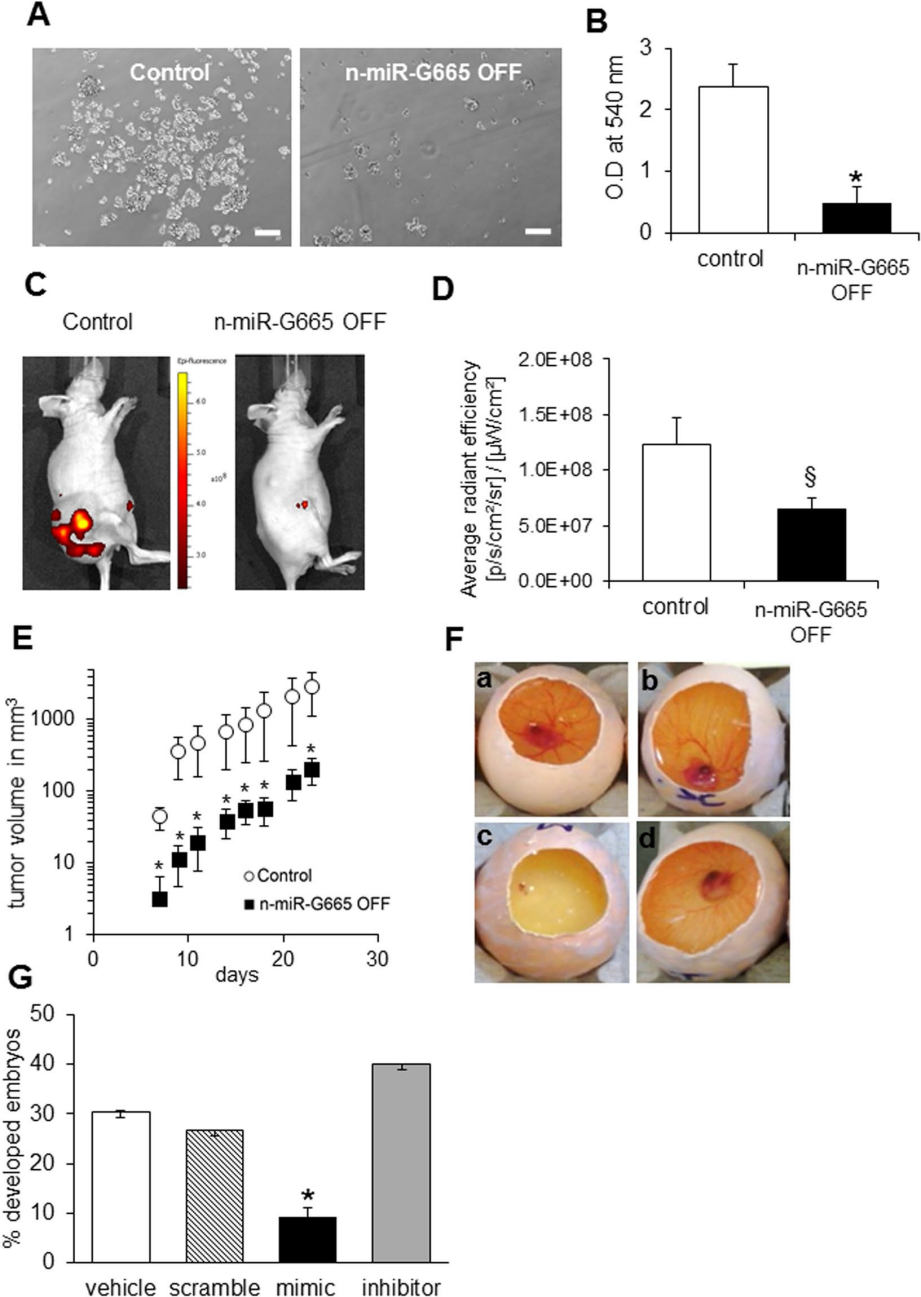
n-miR-G665 inhibition blocks cancer cells sarcospheres formation, chicken embryo growth and impairs tumor growth *in vivo*. (**A**) Representative pictures of sarcospheres obtained after 6 days of culture in non-adherent conditions. (**B**) Semi-quantification of sarcospheres formation by crystal violet staining, *P < 0.01. (**C**) Representative optical imaging acquired in the lateral position of control or n-miR-G665 OFF tumors developed in *nu/nu* mice. (**D**) Quantification of GFP fluorescence activity in regions of interest (ROI) draw free hand in the region of tumor, expressed as the average radiance efficiency, ^§^*P* < 0.05. (**E**) Tumor growth curves of control (open circle) and n-miR-G665 OFF cells in *nu/nu* mice (log scale), *P < 0.01. (**F**) Representative images of embryos developed 6 days post-injection with vehicle (PBS with transfectant) (a), scramble negative control (b), mimic (c) and inhibitor of n-miR-G665 (d). (**G**) Survival rate of chicken embryo injected with n-miR-G665 mimic or inhibitor, *P < 0.01.

The ability of some cancer cells to form spheres reflects their anchorage-independent properties and their dedifferentiated state^22^. We observed that the inhibition of n-miR-G665 impaired the ability of KHOS osteosarcoma cell line to form sarcospheres (Fig. 5A,B). KHOS cells are well known to form fast growing tumor once injected to immunocompromised mice^23^. In xenograft assays, KHOS n-miR-G665 knock out tumors grew at 10-fold slower rate than control tumors (Fig. 5C–E). The data from cancer cell lines and earlier tissue expression studies clearly indicates the association of n-miR-G665 in proliferative cells. Therefore, we asked a question if the expression is higher in embryological tissues that are expected to be proliferating actively and tested it using chicken embryos, a well characterized model to study vertebrate embryology^24^. Three days-old chicken embryos were injected with n-miR-G665 stable mimic sequence (agomiR) which lead to a growth arrest of embryos inducing a dramatic decrease in survival rate (Fig. 5F,G). The data expressed here clearly demonstrates the important and active role of n-miR-G665 in proliferation and potentially in cell cycle.

### n-miR-G665 knock down revealed potential mRNA targets

**Figure 6:**
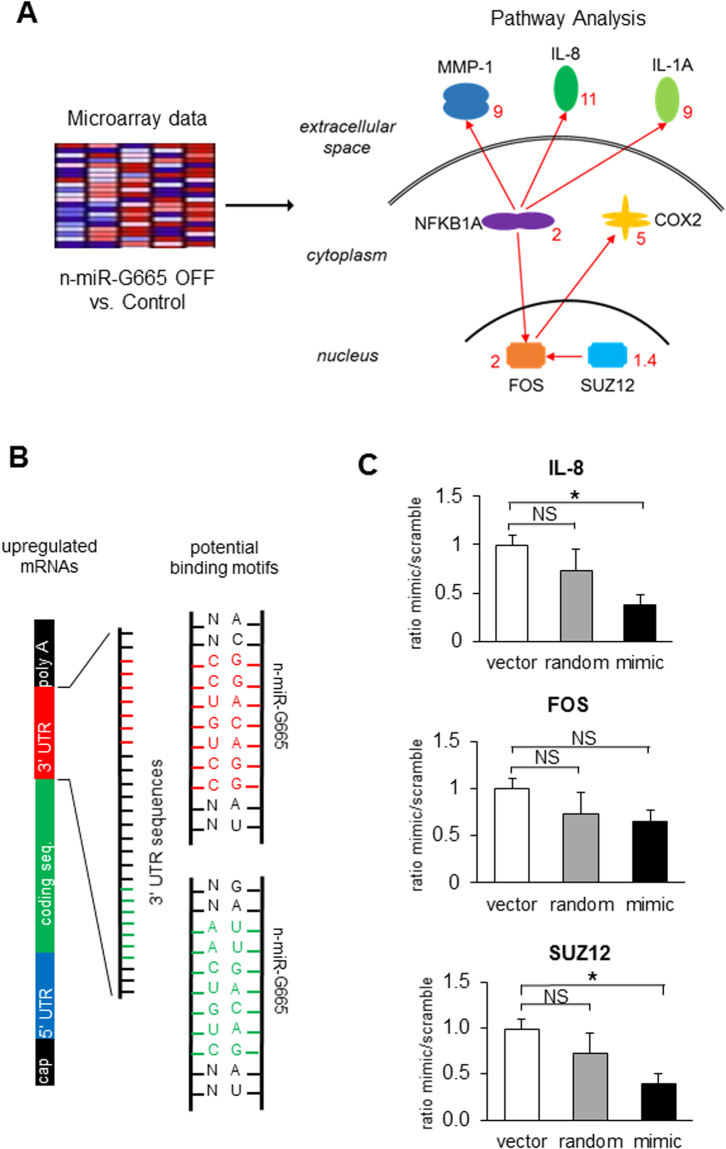
n-miR-G665 potential mRNA targets and binding sites. (**A**) Microarray analysis of genes downregulated in n-miR-G665 OFF cells versus control led to a schematic cellular map of potential interactions between targets (red numbers: fold upregulation mRNA expression in n-miR-G665 OFF cells). (**B**) Schematic representation of n-miR-G665 interaction with 3′UTR potential targets. Potential seed sequences on n-miR-G665 are highlighted in red and green. (**C**) Effect of n-miR-G665 mimic on IL-8, FOS and SUZ12 3′UTR sequences in reporter luciferase activity assays, *P < 0.01.

The functional effects of miRNA are mediated by their mRNA targets. Therefore, we performed a differential gene expression analysis on n-miR-G665 stable knockdown cells, which revealed 92 genes (threshold ≥ 2-fold) upregulated compared to control cells (Supplemental Table S2). Among the most upregulated, we found extracellular proteins like IL-8, IL-1A, MMP-1, intra-cytoplasmic proteins NFκB1A and COX2 and at a less extent intra-nuclear localized proteins like FOS and SUZ12. Ingenuity Pathways analysis illustrated the interrelationship of these specific genes in Fig. 6A. We prospected 3′UTR sequences of 92 mRNAs upregulated in n-miR-G665 knock out cells (see above) to find a seed sequence pattern that could match with n-miR-G665 mature sequence. Based on occurrence results, we identified two potential 7-mer binding motifs on n-miR-G665 mature sequence which have the potential to disrupt target mRNAs stability (see Fig. 6B). To validate bioinformatics analysis, we performed 3′UTR luciferase assay which confirmed the inhibitory role of n-miR-G665 mimic sequence (agomiR) on IL-8 and SUZ12 transcription activity (Fig. 6C).

**Figure 7:**
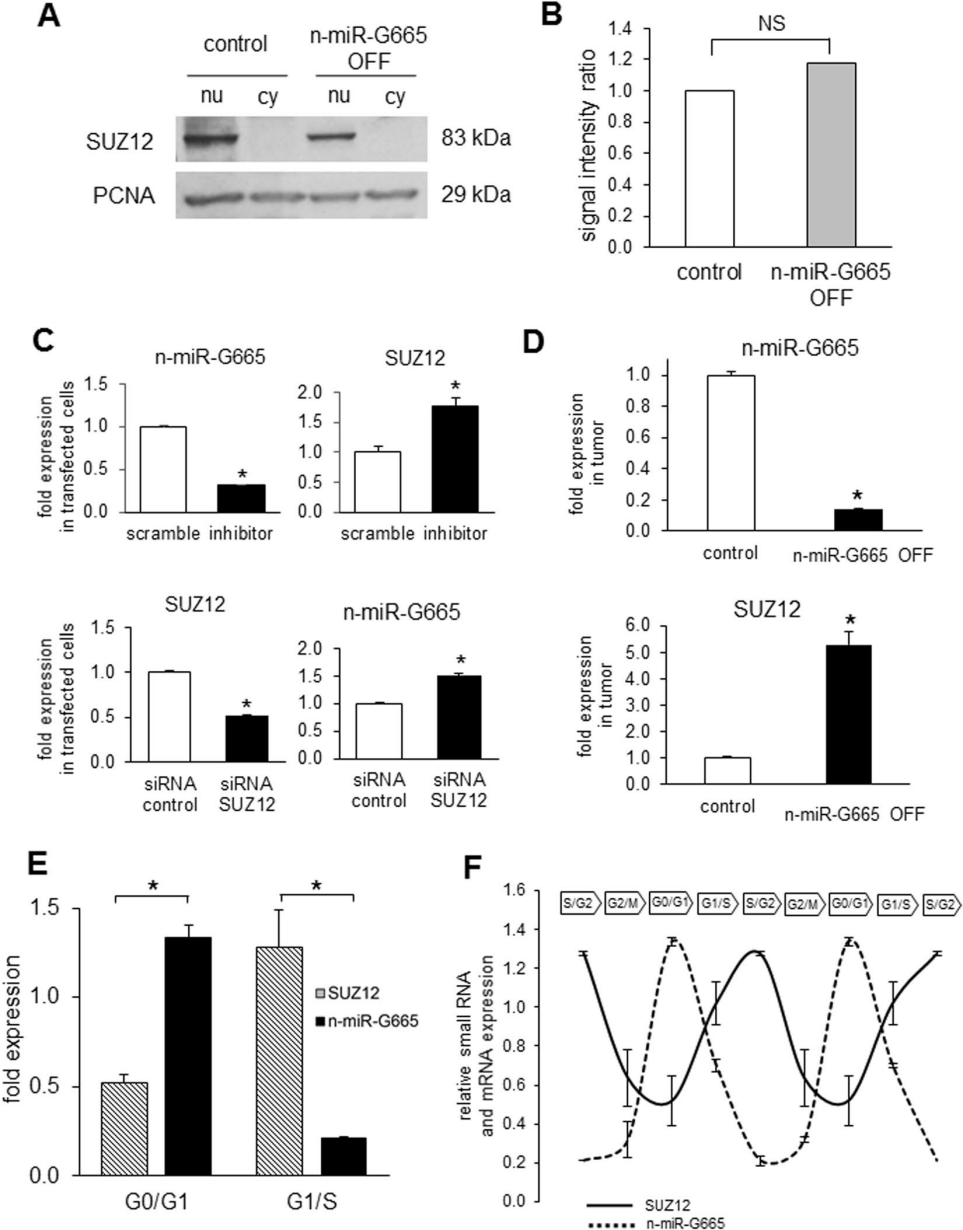
SUZ12 expression at cellular and tissular levels and during cell cycle progression. (**A**) Suz12 protein level expression in nucleic (nu) and cytosolic (cy) fractions in control vector and n-miR-G665 OFF cell lines. (**B**) Densitometry evaluation of SUZ12 protein expression in nucleic fractions (normalized to PCNA), NS: not significant. (**C**) Effect of transient downregulation of n-miR-G665 and SUZ12 on their respective relative levels of expression, *P < 0.01. (**D**) n-miR-G665 and SUZ12 relative levels of expression in tumoral tissue obtained from mice in Fig. 5, *P < 0.01. (**E**) Relative expression levels of SUZ12 and n-miR-G665 in G0/G1 and G1/S phases, and (**F**) through cell cycle progression, *P < 0.01.

Based the role of n-miR-G665 on proliferation and development as demonstrated earlier, we narrowed our study to SUZ12, a polycomb repressor complex 2 component that is a critical in cell proliferation. First, we looked at Suz12 total protein expression in our model of n-miR-G665 knock out cell line (Fig. 7A,B) and did not observe changes. Next, we observed at the miRNA and mRNA levels that transient inhibition of n-miR-G665 induces an increase of SUZ12 mRNA expression. Conversely, the transient inhibition of SUZ12 using siRNA induces an increase of n-miR-G665 expression in transfected cells (Fig. 7C). We measured the relative levels of SUZ12 mRNA levels of expression in mice tumors collected from Fig. 5C and observed that n-miR-G665 knock out tumor samples contains express five times more SUZ12 mRNA levels (Fig. 7C,D). Based on the expression patterns and the inverse correlation between SUZ12 and n-miR-G665, next we explored the possibility of a feedback loop regulation as represented in the schematic shown in Fig. 7D.

### Feedback loop regulation of n-miR-G665 and SUZ12

Furthermore, the expression assays to determine the dosage and expression of n-miR-G665 and SUZ12 demonstrated an inverse correlation with cell cycle stage. The expression levels of n-miR-G665 and SUZ12 fluctuate in opposite directions during the cell cycle, with maximum levels of n-miR-G665 reached during G0/G1 phase and maximum levels of SUZ12 detected during S/G2 phase (Fig. 7E,F and Supplemental Figure S3).

**Figure 8:**
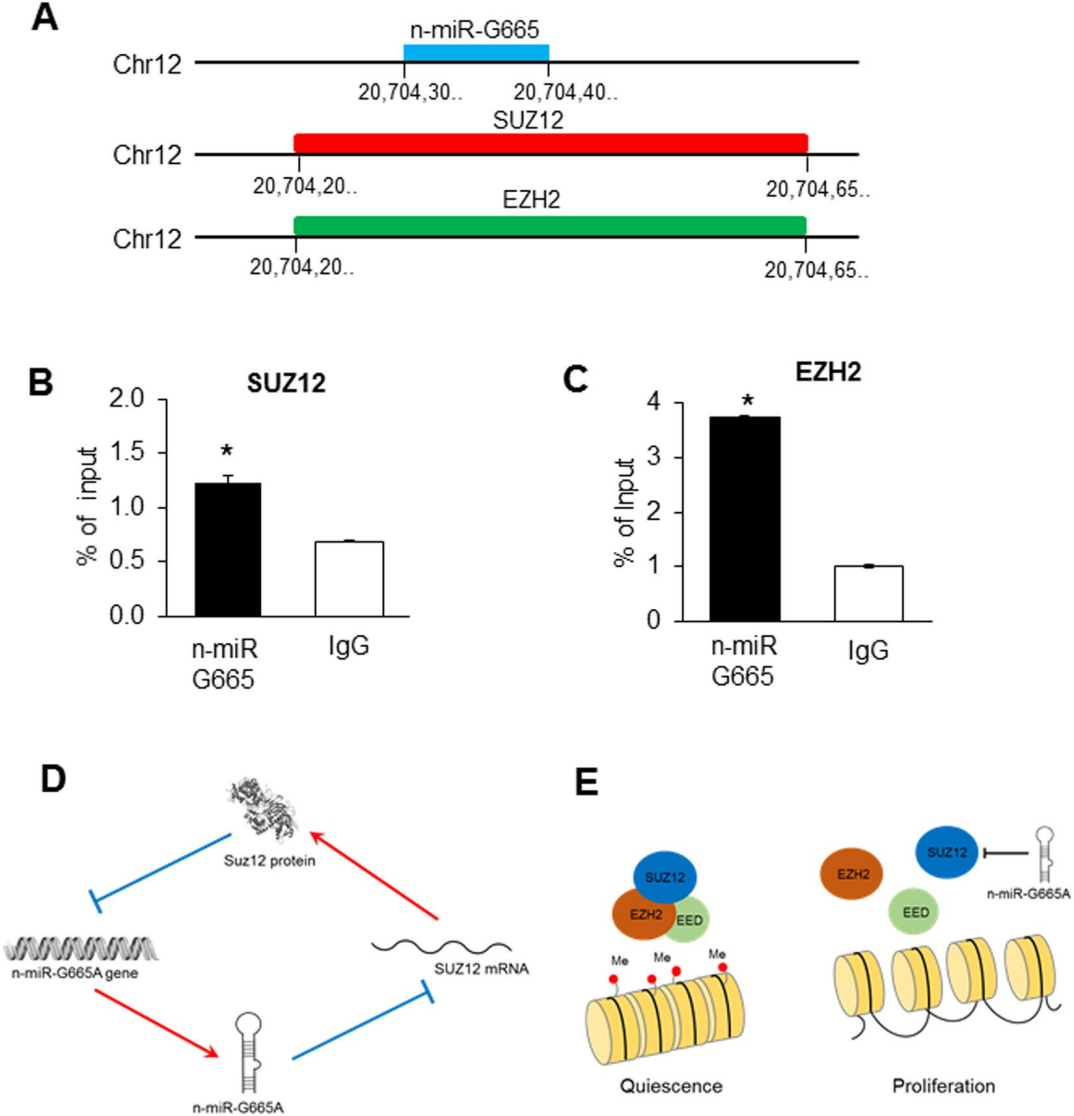
Polycomb Repressor Complex (PRC2) targets n-miR-G665 genomic location. (**A**) Region of binding of transcription factors EZH2 and SUZ12 reported by Transcription Factor ChIP-seq from ENCODE (V2) in GRCh37/hg19 Assembly. (**B**,**C**) Detection of PRC2 complex components binding to miR-G665 genomic DNA region by SUZ12 and EZH2 by chromatin immunoprecipitation (*P < 0.01, compare to IgG). (**D**) Schematic representation showing n-miR-G665 switching cell status from quiescence to proliferation by repressing SUZ12 expression, then PRC2 complex methylation activity. (**E**) Schematic representation of the hypothetical relationship of miR-G665 and SUZ12 (feedback loop mechanism).

The inverse correlation of n-miR-G665 and target gene SUZ12 expression during cell cycle supports the hypothesis of a negative feedback regulatory loop may exist between these two players. To further explore this possibility, we investigated the genomic context of n-miR-G665 DNA sequence which covered a genomic area described in Fig. 1B (Chr12+:20704303-20704437). This region is highly enriched for histone mark chemical modifications (e.g. methylation and acetylation) by H3K27Ac^25^ as demonstrated by the mapping of polycomb repressor complex components SUZ12 and EZH2 binding sites as shown in (Fig. 8A). To confirm the overlap, we used ChIP assays to verify that SUZ12 and EZH2 binds to n-miR-G665 specific coding region using primers that span sequence as showed in Fig. 8B,C.

Data presented in this study demonstrates that the effect of n-miR-G665 is limited to the post-transcriptional level and may represent a regulatory switch for the role of SUZ12 protein (schematic Fig. 8D). As shown in the schematic the feedback loop mechanism allows for stringent regulation of SUZ12 during proliferation, which is required for controlled cell proliferation. n-miR-G665 controls the SUZ12 mRNA expression, modifying the equilibrium of PRC2 complex, and by this action permits the transition of the cell from a quiescent to a proliferation state (schematic Fig. 8E).

## Discussion

Previously, we demonstrated that cargo of hMSC-EVs support tumor growth and an important functional cargo is miRNA^19^, which have the ability to regulate genes involved in multiple cellular processes. Utilizing bioinformatics and previously published studies by others, we identified several known miRNAs of interest and several not annotated microRNA, one of which is n-miR-G665. The functional effect of EVs in tumor growth includes the yet undefined miRNA, it was important to decipher and identify the roles of these yet described novel small RNA.

For a small RNA sequence to be qualified as a microRNA the sequence has to meet several requirements. As a first step towards determining the role of potential miRNA n-miR-G665 is to confirm the phenotype of that miRNA. Towards that we have demonstrated that this n-miR-G665 resembles expression and biogenesis characteristics of miRNA such as (a) ∼22-nt RNA transcript that can be traced back to genomic location - shown in Fig. 1. (b) Such sequences must precisely match the genomic sequence of the organism from which they were identified – shown in Fig. 1. (c) Prediction of a potential fold-back precursor structure that contains the ∼22-nt miRNA sequence within one arm of the hairpin. In this criterion, the hairpin must be the folding alternative with the lowest free energy, as predicted by mfold as shown in Fig. 2. (d) Phylogenetic conservation of the ∼22-nt miRNA sequence and its predicted fold-back precursor secondary structure as shown in Fig. 3, and (e) Detection of increased precursor accumulation in organisms with reduced Dicer function^26^ (Fig. 2C,D). Figures 1, 2 and 3 provide evidence to support all the rules to categorize the novel small RNA as a miRNA^26,27^.

Next step to validate and prove the role of novel small RNA as miRNA is to confirm the functionality of the miRNA, which is to inhibit gene expression. To that end, we performed over expression and knock down studies. As shown in Figs 4 and 5, use of LNA to inhibit this RNA demonstrates its role in cell proliferation demonstrating phenotypic role of n-miRNA G655. This offers strong evidence of novel small RNA to be miRNA, we performed experiments to identify its target genes and the role of this miRNA in cell physiology.

The strategy to identify potential targets involved generating a stably knockdown cell line using an LNA and lentiviral construct lead us to several targets IL-8, FOS and SUZ12. These targets were screened for their GO associations for role in cell proliferation, and SUZ12 is chosen for further studies.

Data from Figs 7 and 8 confirms that n-miR-G655 targets SUZ12 and this correlation overlaps with the cell cycle pattern. Our observations that the n-miR-G655 binds to SUZ12 3′UTR and the Suz12 regulates the expression of n-miR-G655 categorizes this miRNA to belong the family of self-regulatory miRNA^28–30^. Our results confirm that n-miR-G665 small RNA sequence is indeed a miRNA that is highly expressed in EVs secreted by MSCs is novel miRNA that targets SUZ12.

## Materials and Methods

### Cell culture

KHOS, U2OS, MG63, HUVEC, HT1080 were obtained from ATCC (Manassas, VA). MCF7 and MDA-MB 231 breast cancer cell lines were a generous gift from Dr. Brian G. Rowan, Tulane University, New Orleans, LA. HUVEC and HEK293T cell lines were a generous gift of Dr. Joseph Maher, University of Mississippi Medical Center, Jackson, MS. All cancer cell lines were maintained in culture with Dulbecco’s Minimum essential medium (DMEM, Gibco-BRL), 10% fetal bovine serum (FBS, Atlanta Biologicals, Inc.), 100 units/ml penicillin, 100 µg/ml streptomycin. Mesenchymal stem cells (MSCs) were obtained from the National Institutes of Health-funded MSC distribution center (medicine.tamhsc.edu/irm/msc-distribution.html) at the Texas A&M Health Science Center College of Medicine Institute for Regenerative Medicine (Temple, TX) in accordance with guidelines and regulations of their institutional committee. Informed consent was obtained from all subjects. MSCs from two healthy young male donors were used for this study, from frozen vials obtained at passage 1. MSCs were cultured until reaching 80% confluency from passage 2 to passage 4. MSCs were cultured in alpha-MEM supplemented with 17.5% of FBS and (Atlanta Biologicals, Flowery Branch, GA), 100 units/ml penicillin, 100 µg/ml streptomycin. shDrosha hMSCs cell model have been previously described and the Dicer knockout MCF7 breast cancer cells model have been obtained using the same protocol described in^21^.

### Extracellular vesicles (EVs) isolation

EVs were isolated using a protocol described in^19^. Briefly, MSCs were cultured in alpha-MEM supplemented with 17.5% FBS until 80% confluency in two-layered cell factories (Nunc, ThermoFisher) (approximately 30 million total cells). Culture medium was replaced with serum free medium (plain alpha-MEM) and cells were grown under these serum-deprived conditions for a period up to 30 days. Cell supernatant was collected every 3 days and centrifuged at 500 g for 10 min to eliminate cell debris. The supernatant was concentrated using a N2 positive pressure concentrator (Amicon 8400) with 1 kDa ultra-filtration discs (Millipore) to a final volume of 5 ml and ultra-centrifuged at 15,000 g for 1 hour at 4 °C to remove large vesicles. The supernatant which contain the extracellular vesicles (EVs) fraction was further subjected to ultracentrifugation at 110,000 g for 18 hours at 4 °C. EVs pellets were washed with PBS and ultracentrifuged again at 110,000 g for 18 hours at 4 °C. Pellets obtained were re-suspended in 50 to100 μL of PBS, and aliquots were stored at −80 °C. To verify purified EVs preparations quality, a fraction of aliquots are processed to electron microscopy (Microscopy Core, University of Mississippi Medical Center, Jackson, MS) and assayed for protein contents (micro bicinchoninic assay, Pierce-ThermoFisher, Whaltham, MA).

### Next generation sequencing

Next generation sequencing has been outsourced to the microRNA sequencing service provided by LC Sciences LLC (Houston, TX). Briefly, a small RNA library was generated from EVs that are secreted from two normal healthy MSCs donors (see EVs isolation description above) using the Illumina Truseq small RNA preparation kit according to the manufacturer protocol (Illumina, San Diego, CA). The purified cDNA library was used for cluster generation on Illumina’s cluster station and then sequenced on Illumina GAIIx. Raw sequence reads, up to 40 nucleotides, were obtained using Illumina’s sequencing control studio software version 2.8 following real-time sequencing image analysis and base-calling by Illumina’s real time analysis software version 1.8.70. Analysis of sequencing data were performed using LC Sciences proprietary pipeline script ACGT101-miR version 4.2. Around 15 million of raw reads were filtered and categorized in different groups as described in Supplemental Figure S1. Reads unmapped to selected miRs in miRbase, unmapped to mRNA, Rfam and repBase but mapped to species specific genome and for which the extended sequences could potentially form hairpins were selected as potential new miRNAs.

### PCR for DNA mapping

hMSCs cells DNA were extracted using PureLink Genomic DNA Mini Kit (LIfeTech). 10 ng were mixed with the different 100 nM set of primers (see primers list in Supplemental Methods) and with PCR Mastermix containing the Taq DNA polymerase, dNTPs and all other components (ThermoFisher, cat#K0171) following manufacturer recommendation. PCR products were resolved on 2% agarose gel and visualized with Biorad Universal hood II imaging station.

### Real time PCR for miRNA detection

Total or small RNA fraction of EVs and cells were isolated using miRVANA isolation kit (Ambion). 5 ng of RNA samples were converted to cDNA using a TaqMan MicroRNA Reverse Transcription Kit (Applied Biosystems, cat# 4366596,) according to the manufacturer’s instructions. Real-time PCR was performed using TaqMan Universal PCR Master Mix (cat# 4324018, Applied Biosystems) and TaqMan Assay miRNA Mix (Applied Biosystems) as recommend by the manufacturer. A list of RT and PCR probes used is provided in Table S1.

#### Sequence alignment and radial phylogenic tree

Sequences from multiple species were retrieved using UCSC Genome Browser^31^ on Human May 2004 (NCBI35/hg17) Assembly and aligned using Jalview version 2.9.0b2 software (see Supplemental Figure S1)^32^. The radial phylogenic tree has been designed using the T-Rex webserver^33^.

### Transient siRNA transfection

Transient downregulation of n-miR-G665 were obtained using Locked Nucleic Acid inhibitor sequence from Qiagen (Germantown, MD). Transient downregulation of SUZ12 were obtained using. HT1080 or KHOS cells were seeded at 50,000 cells/cm2 in 6-well plate and transfected the next day with 12 μL of HiPerfect transfection reagent (Qiagen) in presence of 100 pmol of n-miR-G665 LNA inhibitor sequence or a scramble sequence (AllStars Negative control AF555, Qiagen cat# 1027286) or 100 pmol of SUZ siRNA (Qiagen, cat#AM16708) or a negative siRNA control (Qiagen, cat#AM4611). Cells were incubated overnight and then assayed for cell proliferation by measuring DNA content using a Cyquant assay (Invitrogen) or assayed to evaluate the level of n-miR-G665 or SUZ12 mRNA levels of expression by RT-qPCR.

### Lentivirus transduction

Lentiviral constructs were obtained from Applied Biological Materials (Richmond, BC, Canada) and permanent cell lines were obtained per manufacturer’s protocol. Briefly, KHOS, MDA-MB231 and HEK293T were transfected with 10 μg of pLenti-miROFF-III-GFP-puro vector (containing the n-miR-G665 target sequence or a control sequence, see Fig. 4B) with lipofectamine. Transduced cells were selected and maintained in culture with puromycin at 1 μg/mL. Cells were further selected by clonal selection and were selected based on their n-miR-G665 downregulation levels.

### Cell proliferation assay

10,000 cells per well were plated in 96-well plate format and DNA content were quantified every day for 10 days using a Cyquant assay (Invitrogen, CA).

### Sarcospheres assay

Single cell suspensions were collected and 5 × 103 cells were plated in each well of a Nunc Low-Cell Binding (Nunc, Rochester, NY) six-well plate. Cells were incubated for 12 days before being transferred to adherent plates to allow for adherence for 24 hours. Colonies were then stained with Crystal Violet solution (Sigma-Aldrich) and colonies containing more than 200 cells were quantified. Clonal density was used as described by Patrawala *et al*.^34^ and non-adherent plates were used as substitutes for agar plating.

### *In vivo* mouse experiments

NU/NU female mice were purchased from Charles River Laboratories (Wilmington, MA). All animal studies were conducted in accordance with NIH animal use guidelines and a protocol approved by UMMC Animal Care Committee. Nude mice were injected subcutaneously with 1 million cells of KHOS control or KHOS miROFF and tumor progression were monitored during 23 days. Tumor volumes were calculated using the formula V = (4/3) π*a*^*2*^*b*, where a is shorter radius in mm and b is longer radius in mm. At endpoint, bioluminescence (GFP) imaging is conducted using IVIS Xenogen bioimager (Caliper, Perkin Elmer, Waltham, MA).

### Chicken embryo viability assay

Fertilized day 1 chicken eggs were obtained from Tyson Inc. Hatchery (Magee, MS). 50 μM of n-miR-G665 lock acid nucleic mimic, inhibitor (see Table S1) or scramble (negative control B, Exiqon) in presence of 0.24 μL of HiPerfect transfection reagent (Qiagen) and diluted in 20 μL of sterile PBS buffer were injected in the center of the embryonic disc using a 28G1/2 (0.36 mm × 13 mm) insulin syringe (Beckton Dickinson Franklin Lakes, NJ). Control PBS and HiPerfect were performed using the same procedure. The embryos were harvested at day 6 post-injection for viability and classified as fully developed or undeveloped.

### Microarray

RNA was isolated from KHOS miROFF n-miR-G665 and KHOS miROFF Control cells and evaluated for quality and integrity (Bio-Rad Experion™ System). Whole genome transcript analysis was performed using Affymetrix 3000 7 G System platform using Human GeneChip 2.0ST arrays covering >30,000 coding transcripts. RNA samples were processed per manufacturers’ protocols using Ambion Whole-Transcript (WT) Expression kit, and Affymetrix GeneChip WT Terminal Labeling and Controls Reagent kit on Affymetrix equipment (Scanner 3000 7 G System). Hybridized chips processed at the UMMC Institutional Molecular and Genomics Core.

### Potential 7-mer seed binding sites

Based on the 3′UTR sequence of 48 mRNAs upregulated (threshold ≥ 3-fold) in KHOS n-miR-G665 knock out cells, we look on potential 7-mer pair match with the 19 nucleotides n-miR-G665 mature sequence (Fig. 1E) by using a computerized program developed in-house by the UMMC Bioinformatics core. All potential matching positions were collected and 7-mer seed sequences which scored the most occurrence.

### 3′UTR luciferase assay

This assay was performed per manufacturer’s protocol (SwitchGear Genomics, Carlsbad, CA). Briefly, HT1080 fibrosarcoma cells were transfected in presence of DharmaFect (GE Dharmacon, Lafayette, CO) with a GoClone Luciferase reporter gene containing the full 3′UTR sequence of SUZ12 (accession number NM_015355) in presence or not of n-miR-G665 locked nucleic acid mimic sequence (Qiagen) or a control scramble (All Star Neg siRNA, Qiagen) sequence (see Table S1). After an overnight incubation, the luciferase activity was measured using LightSwitch assay reagent kit (SwitchGear Genomics) and a plate luminometer reader (BioTek, Synergy4). Results are expressed at the ratio of signal obtain by the n-miR-G66A mimic compared to the scramble sequence.

### Western blot

Proteins were extracted and fractionated by SDS-PAGE as previously described 7. Briefly, nuclear and cytoplasmic fractions were extracted using a NE-PER kit per manufacturer recommendations (Pierce Rockford, IL), protein contents estimated using BCA assay (Pierce). Western blot assays were performed using 15 µg of protein samples, resolved on 4–12% NU-PAGE Bis-Tris acrylamide gel (Invitrogen) and proteins transferred on a PVDF membrane (GenHunter, Nashville, TN) and subjected to Suz12 (Millipore, cat#17661, 1:1000 dilution) or PCNA (AbCam cat#18197, 1:1000 dilution) detection. We used secondary antibody goat anti-mouse or rabbit conjugated with HRP (Abcam, cat# ab6789 and ab6721 respectively) diluted 1:10,000. Once incubated with enhanced chemiluminescent substrate (Pierce), CL-Xposure films (Thermo Scientific) were exposed and revealed using a Kodak M35-A X-OMAT processor. Band intensity was evaluated by ImageJ software.

### Cell cycle assay

Ten million KHOS cells were incubated 18 hrs with thymidine 2 mM, etoposide 5 μM, vinblastine 5 nM or without serum to block cells in G1/S, S/G2, G2/M and G0/G1 phases respectively^35^. Cells were trypsinized, fixed in ethanol, treated with RNAse A and stained with propidium iodide. Cells were analyzed using a Gallios flow cytometer (Beckman-Coulter) at the UMMC Flow cytometry Core. Cell cycle profiles were validated (see Supplemental Figure S3) before SUZ12 and n-miR-G665 expression levels were measured by quantitative real-time PCR (see above).

### ChIP assay

ChIP assay was conducted according manufacturer protocol (EZ ChIP chromatin immunoprecipitation kit, Upstate/Millipore). Briefly, 30 million KHOS cells were crosslinked with fresh prepared 1% formaldehyde for 10 min at room temperature then quenched with glycine for 5 min. Cells were scraped in cold PBS and protease inhibitor cocktail then centrifuged and cell pellets were re-suspended in SDS lysis buffer containing a protease inhibitor cocktail. DNA was sheared using a sonicator-dismembrator (Fisher Scientific, model 100, 5 strokes at power 2 for 10 sec, 1 min iced rest between) on wet ice and insoluble material was removed by centrifugation. Sheared crosslinked chromatin were pre-incubated with protein G agarose per manufacturer’s recommendation then mix with antibodies of interest (anti-SUZ12 or anti-EZH2). A positive control (anti-RNA polymerase) and negative control (normal mouse IgG) were also included in the experiment. After an overnight incubation antibody/antigen complex were collected by incubation with protein G agarose and washed with low and high salt immune complex buffer, LiCl immune complex buffer then Tris EDTA buffer. Antibody/ antigen complex bound to protein G agarose beads were eluted using SDS/NaHCO3 buffer, and the free DNA were obtained by reverse crosslinking using overnight incubation at 65 °C with NaCl, RNAse A and proteinase K treatment. Free DNA obtained were purified with columns per manufacturer’s recommendation and subjected to PCR using primers (see Table S1).

### Statistical analysis

The data are represented as average ± standard error of the samples. All experiments were performed three times. Statistical analysis was performed using non-paired t-test, and p value < 0.05 was considered statistically significant.

### Data availability

The datasets generated during and/or analyzed during the current study are available from the corresponding author on request.

## Electronic supplementary material

Supplemental data
