# Supplementary material for: Polycomb group protein Suz12 is regulated by a novel miRNA-like small RNA
Source: Sci Rep. 2018 Jan 29;8:1720. doi: 10.1038/s41598-018-19989-5 (PMC5788869; doi:10.1038/s41598-018-19989-5)
Supplement: Supplementary file 1 — Supplemental data [file 41598_2018_19989_MOESM1_ESM.pdf]

SUPPLEMENTARY INFORMATION

Title: Polycomb group protein Suz12 is regulated by a novel miRNA-like small RNA.

Authors: Patrice Penfornis[1], Joseph D. Fernandes[1] and Radhika R. Pochampally[1, 2 *]

Supplemental Figure Legends

Table S1: List of primers, agomiR and antagomiR used in the study.

Table S2: List of genes upregulated in cells expressing low levels of n-miR-G665.

Supplemental Figure S1: Flowchart of data analysis provided by LC Sciences, LLC (Houston, TX).

Supplemental Figure S2: Sequence alignment of n-miR-G665 in UCSC Genome Browser on Human May 2004 (NCBI35/hg17) Assembly.

Supplemental Figure S3: Representative propidium iodide plots obtained by flow cytometry after treatment with respective drugs.

Table S1

PCR Primers list (provider: IDT DNA)

PDE3A gene	Forward primer	Reverse primer
Set A	5’-AGCCAGATCAATTTGTCCTCTT-3’	5’-GTCGCACTCATCGCACAT-3’
Set B	5’-AAAGATTTACGGACAGGATTGAC-3’	5’-ATGTGGAAGGCCATGTCG-3’
Set C	5’-GTTCTCACTCTTCCCTCAGTTT-3’	5’-GCCTTCTTCTTGCTCTCTCTC-3’

RT-PCR Primers list (provider: Applied Biosystems – ThermoFisher)

Gene name	Catalog number
n-miR-G665	CUSTOM #CSOIWAE
RNU6B	PN4427975
IL-8	Hs00174103_m1
FOS	Hs04194186_s1
SUZ12	Hs00248742_m1
5S	Hs03682751_gH

AgomiR and antagomiR (provider: Qiagen)

Type	Catalog #	Lot #
n-miR-G665 agomiR (mimic)	CUSTOM	188897805
n-miR-G665 antagomiR (inhibitor)	CUSTOM	116404230
All Stars Neg siRNA scramble	1027286	113176935

Table S2

Pairwise Analysis: HuGene ST 2.0

	**Group 1 KHOS miROFF Control**	**Group 2 KHOS n-miR-G665 A3**		
**No.**		**Ratio**	**Identifier**	**Gene Name**
1	up-regulated	11.01	NM_000584	Interleukin 8
2	up-regulated	8.95	NM_002421	Matrix metallopeptidase 1 (interstitial collagenase)
3	up-regulated	8.48	NM_000575	Interleukin 1, alpha
4	up-regulated	6.55	NM_000576	Interleukin 1, beta
5	up-regulated	6.27	NM_004079	Cathepsin S
6	up-regulated	5.63	NM_000758	Colony stimulating factor 2 (granulocyte-macrophage)
7	up-regulated	5.08	NM_001165	Baculoviral IAP repeat-containing 3
8	up-regulated	5.08	NM_032413	Chromosome 15 open reading frame 48
9	up-regulated	5.08	NM_000963	Prostaglandin-endoperoxide synthase 2
10	up-regulated	4.28	NM_004417	Dual specificity phosphatase 1
11	up-regulated	4.16	NM_004994	Matrix metallopeptidase 9
12	up-regulated	4.09	NM_001005239	Olfactory receptor, family 11, subfamily H, member 1
13	up-regulated	4.06	NM_001005484	Olfactory receptor, family 4, subfamily F, member 5
14	up-regulated	3.94	NM_172239	REX1, RNA exonuclease 1 homolog (S. cerevisiae)-like 1
15	up-regulated	3.62	NM_001174166	solute carrier family 16, member 6
16	up-regulated	3.4	NM_006290	Tumor necrosis factor, alpha-induced protein 3
17	up-regulated	3	NM_001017402	Laminin, beta 3
18	up-regulated	3	NM_000594	Tumor necrosis factor (TNF superfamily, member 2)
19	up-regulated	2.98	NM_001004306	Coiled-coil domain containing 144 family, N-terminal like
20	up-regulated	2.84	NM_012328	DnaJ (Hsp40) homolog, subfamily B, member 9
21	up-regulated	2.74	NM_003528	Histone cluster 2, H2be
22	up-regulated	2.71	NM_004056	Carbonic anhydrase VIII
23	up-regulated	2.7	NM_017870	Transmembrane protein 132A
24	up-regulated	2.68	NM_001004727	Olfactory receptor, family 4, subfamily X, member 2

25	up-regulated	2.65	NM_021101	Claudin 1
26	up-regulated	2.62	NM_001097613	G protein-coupled receptor 89A
27	up-regulated	2.57	NM_001945	Heparin-binding EGF-like growth factor
28	up-regulated	2.54	NM_001005514	Olfactory receptor, family 5, subfamily H, member 14
29	up-regulated	2.52	NM_016617	Ubiquitin-fold modifier 1
30	up-regulated	2.51	NM_001964	Early growth response 1
31	up-regulated	2.49	NM_001004685	Olfactory receptor, family 2, subfamily F, member 2
32	up-regulated	2.48	NM_001001674	Olfactory receptor, family 4, subfamily F, member 15
33	up-regulated	2.45	NM_001511	Chemokine (C-X-C motif) ligand 1
34	up-regulated	2.45	NM_006734	HIV type I enhancer binding protein 2
35	up-regulated	2.43	NM_005542	Insulin induced gene 1
36	up-regulated	2.41	NM_005261	GTP binding protein overexpressed in skeletal muscle
37	up-regulated	2.41	NM_005635	Synovial sarcoma, X breakpoint 1
38	up-regulated	2.4	NM_021199	Sulfide quinone reductase-like (yeast)
39	up-regulated	2.39	NM_001561	Tumor necrosis factor receptor superfamily, member 9
40	up-regulated	2.36	NM_033390	Zinc finger CCCH-type containing 12C
41	up-regulated	2.33	NM_000212	Integrin, beta 3 (platelet glycoprotein IIIa, antigen CD61)
42	up-regulated	2.33	NM_002357	MAX dimerization protein 1
43	up-regulated	2.33	NM_031419	Nuclear factor of kappa light polypeptide gene enhancer
44	up-regulated	2.31	NM_152342	Chromodomain protein, Y-like 2
45	up-regulated	2.31	NM_001007249	Olfactory receptor, family 8, subfamily G, member 2
46	up-regulated	2.3	NM_181785	Solute carrier family 46, member 3
47	up-regulated	2.29	NM_005252	V-fos FBJ murine osteosarcoma viral oncogene homolog
48	up-regulated	2.28	NM_016234	Acyl-CoA synthetase long-chain family member 5
49	up-regulated	2.28	NM_001433	Endoplasmic reticulum to nucleus signaling 1
50	up-regulated	2.27	NM_003821	Receptor-interacting serine-threonine kinase 2
51	up-regulated	2.22	NM_001570	Interleukin-1 receptor-associated kinase 2
52	up-regulated	2.22	NM_001005500	Olfactory receptor, family 4, subfamily M, member 1
53	up-regulated	2.21	NM_020307	Cyclin L1
54	up-regulated	2.21	NM_001005200	Olfactory receptor, family 8, subfamily H, member 2
55	up-regulated	2.21	NM_001003799	TCR gamma alternate reading frame protein

56	up-regulated	2.2	NM_001190790	CMT1A duplicated region transcript 15-like 2
57	up-regulated	2.2	NM_018689	KIAA1199
58	up-regulated	2.2	NM_020529	Nuclear factor of kappa light polypeptide gene enhancer
59	up-regulated	2.19	NM_000201	Intercellular adhesion molecule 1
60	up-regulated	2.19	NM_001004760	Olfactory receptor, family 51, subfamily V, member 1
61	up-regulated	2.19	NM_005424	Tyrosine kinase with Ig-like and EGF-like domains 1
62	up-regulated	2.18	NM_152405	Junction mediating and regulatory protein, p53 cofactor
63	up-regulated	2.16	NM_001001916	Olfactory receptor, family 52, subfamily J, member 3
64	up-regulated	2.14	NM_005346	Heat shock 70kDa protein 1A
65	up-regulated	2.14	NM_178354	Late cornified envelope 1F
66	up-regulated	2.14	NM_178230	Peptidylprolyl isomerase A (cyclophilin A)-like 4A
67	up-regulated	2.12	NM_002309	Leukemia inhibitory factor (cholinergic differentiation factor)
68	up-regulated	2.11	NM_004038	Amylase, alpha 1A (salivary)
69	up-regulated	2.11	NM_005345	Heat shock 70kDa protein 1A
70	up-regulated	2.11	NM_031914	Synaptotagmin XVI
71	up-regulated	2.1	NM_152751	BEN domain containing 7
72	up-regulated	2.1	NM_054107	Olfactory receptor, family 1, subfamily J, member 2
73	up-regulated	2.09	NM_005512	Leucine rich repeat containing 32
74	up-regulated	2.09	NM_003247	Thrombospondin 2
75	up-regulated	2.09	NM_006528	Tissue factor pathway inhibitor 2
76	up-regulated	2.08	NM_001706	B-cell CLL/lymphoma 6
77	up-regulated	2.08	NM_153218	Chromosome 13 open reading frame 31
78	up-regulated	2.08	NM_000600	Interleukin 6 (interferon, beta 2)
79	up-regulated	2.08	NM_178429	Late cornified envelope 2C
80	up-regulated	2.07	NM_017655	GIPC PDZ domain containing family, member 2
81	up-regulated	2.07	NM_178430	Late cornified envelope 2D
82	up-regulated	2.06	NM_032148	Solute carrier family 41, member 2
83	up-regulated	2.05	NM_013437	Low density lipoprotein-related protein 12
84	up-regulated	2.05	NM_001005338	Olfactory receptor, family 5, subfamily H, member 1
85	up-regulated	2.04	NM_001159323	phospholipase A2, group IVC
86	up-regulated	2.04	NM_025079	Zinc finger CCCH-type containing 12A

87	up-regulated	2.03	NM_017954	Ca++-dependent secretion activator 2
88	up-regulated	2.02	NM_004038	Amylase, alpha 1A (salivary)
89	up-regulated	2.02	NM_005194	CCAAT/enhancer binding protein (C/EBP), beta
90	up-regulated	2.02	NM_001004744	Olfactory receptor, family 5, subfamily R, member 1
91	up-regulated	2.01	NM_030952	NUAK family, SNF1-like kinase, 2
92	up-regulated	2	NM_000361	Thrombomodulin

©Copyright 2013 PerkinElmer, Inc. All rights reserved.

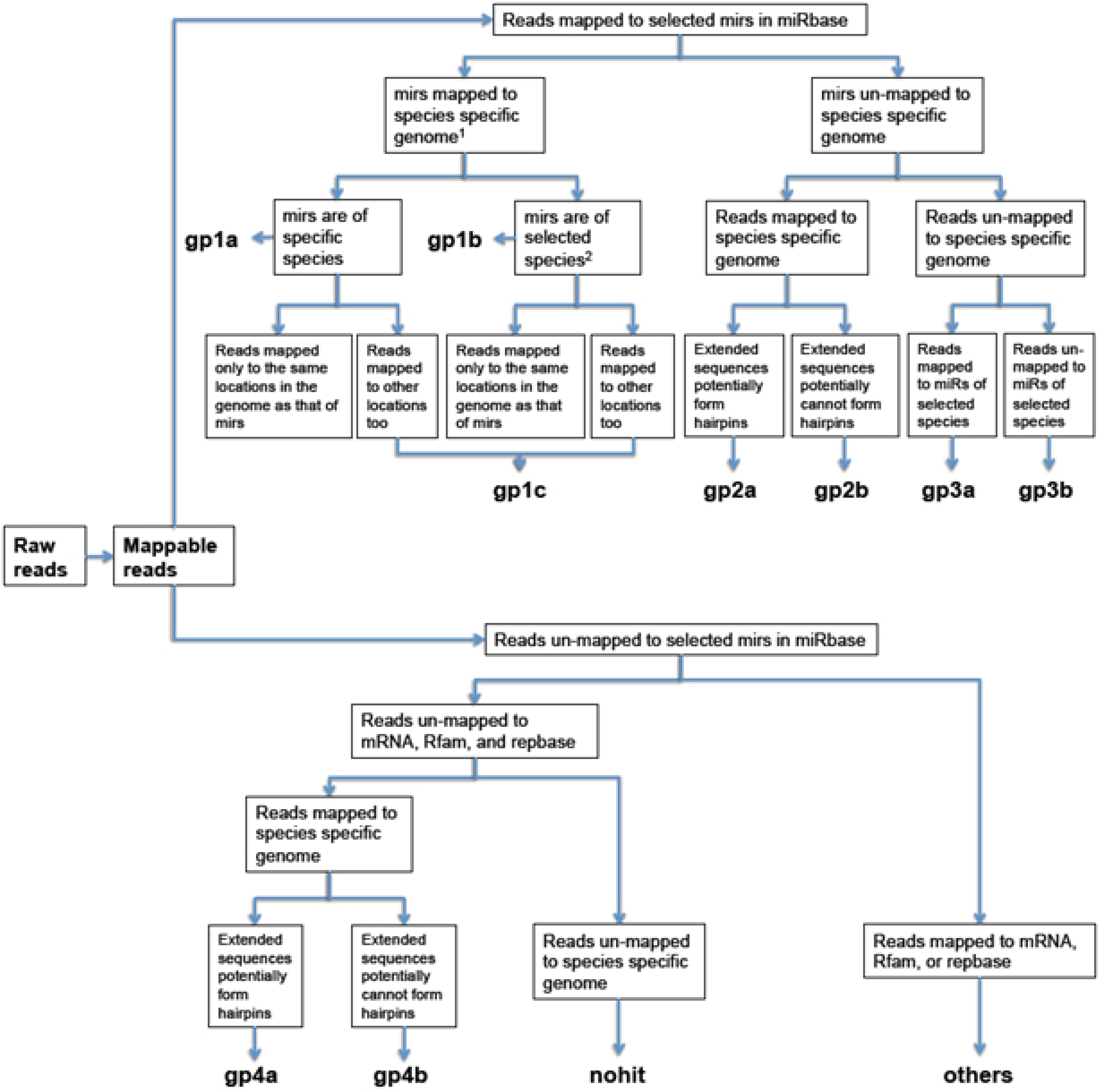

Figure S1: Flowchart of data analysis provided by LC Sciences, LLC (Houston, TX)

A

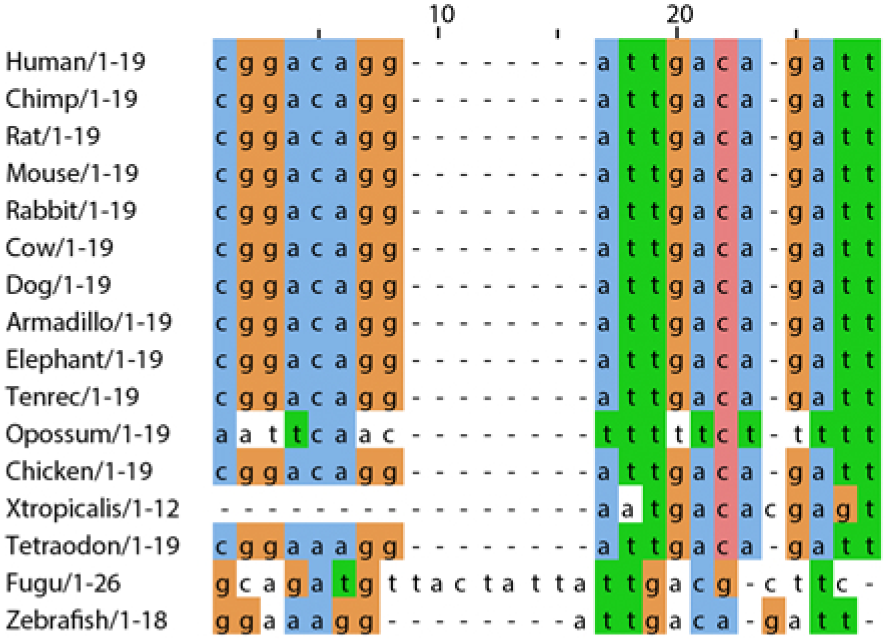

Figure S2: sequence alignment of n-miR-G665 in UCSC Genome Browser on Human May 2004 (NCBI35/hg17) Assembly.

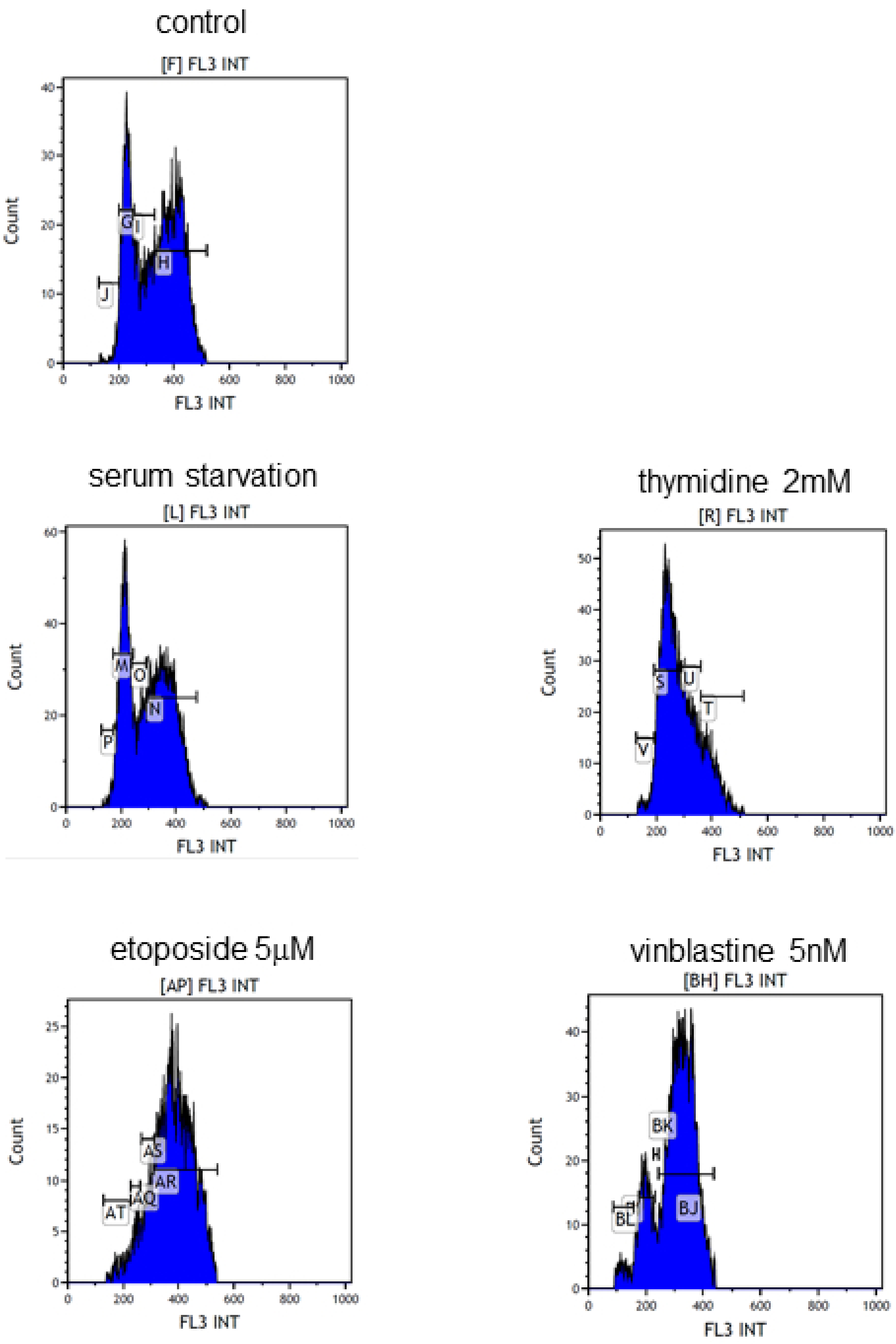

Figure S3: Representative propidium iodide plots obtained by flow cytometry after treatment with respective drugs..
